# A Deep Learning Approach for Classifying Developmental Stages of *Ixodes ricinus* Ticks on Images Captured Using a Microscope’s High-Resolution CMOS Sensor

**DOI:** 10.3390/s25165038

**Published:** 2025-08-14

**Authors:** Aleksandra Marzec, Anna Filipowska, Oliwia Humeniuk, Wojciech Filipowski, Paweł Raif

**Affiliations:** 1 Foundation of Cardiac Surgery Development, Institute of Heart Prostheses, 345a Wolności, 41-800 Zabrze, Poland; aleksandra.marzec-przyszlak@polsl.pl; 2 Department of Medical Informatics and Artificial Intelligence, Faculty of Biomedical Engineering, Silesian University of Technology, Roosevelta 40, 41-800 Zabrze, Poland; anna.filipowska@polsl.pl (A.F.); oliwhum809@student.polsl.pl (O.H.); pawel.raif@polsl.pl (P.R.); 3 Department of Telecommunications and Teleinformatics, Faculty of Automatic Control, Electronics and Computer Science, Silesian University of Technology, Akademicka 16, 44-100 Gliwice, Poland

**Keywords:** *Ixodes ricinus*, tick development stages, deep learning, image classification, CNNs, Grad-CAM, Explainable AI (XAI)

## Abstract

This article presents a deep learning approach for classifying the developmental stages (larvae, nymphs, adult females, and adult males) of *Ixodes ricinus* ticks, the most common tick species in Europe and a major vector of tick-borne pathogens, including *Borrelia burgdorferi*, *Anaplasma phagocytophilum*, and tick-borne encephalitis virus (TBEV). Each developmental stage plays a different role in disease transmission, with nymphs considered the most epidemiologically relevant stage due to their small size and high prevalence. We developed a convolutional neural network (CNN) model trained on a dataset of microscopic tick images collected in the area of Upper Silesia, Poland. Grad-CAM, an XAI technique, was used to identify the regions of the image that most influenced the model’s decisions. This work is the first to utilize a CNN model for the identification of European tick fauna stages. Compared to existing solutions focused on North American tick species, our model addresses the specific challenge of distinguishing developmental stages within *I. ricinus*. This solution has the potential to be a valuable tool in entomology, healthcare, and tick-borne disease management.

## 1. The Problem and Its Background

**Figure 1:**
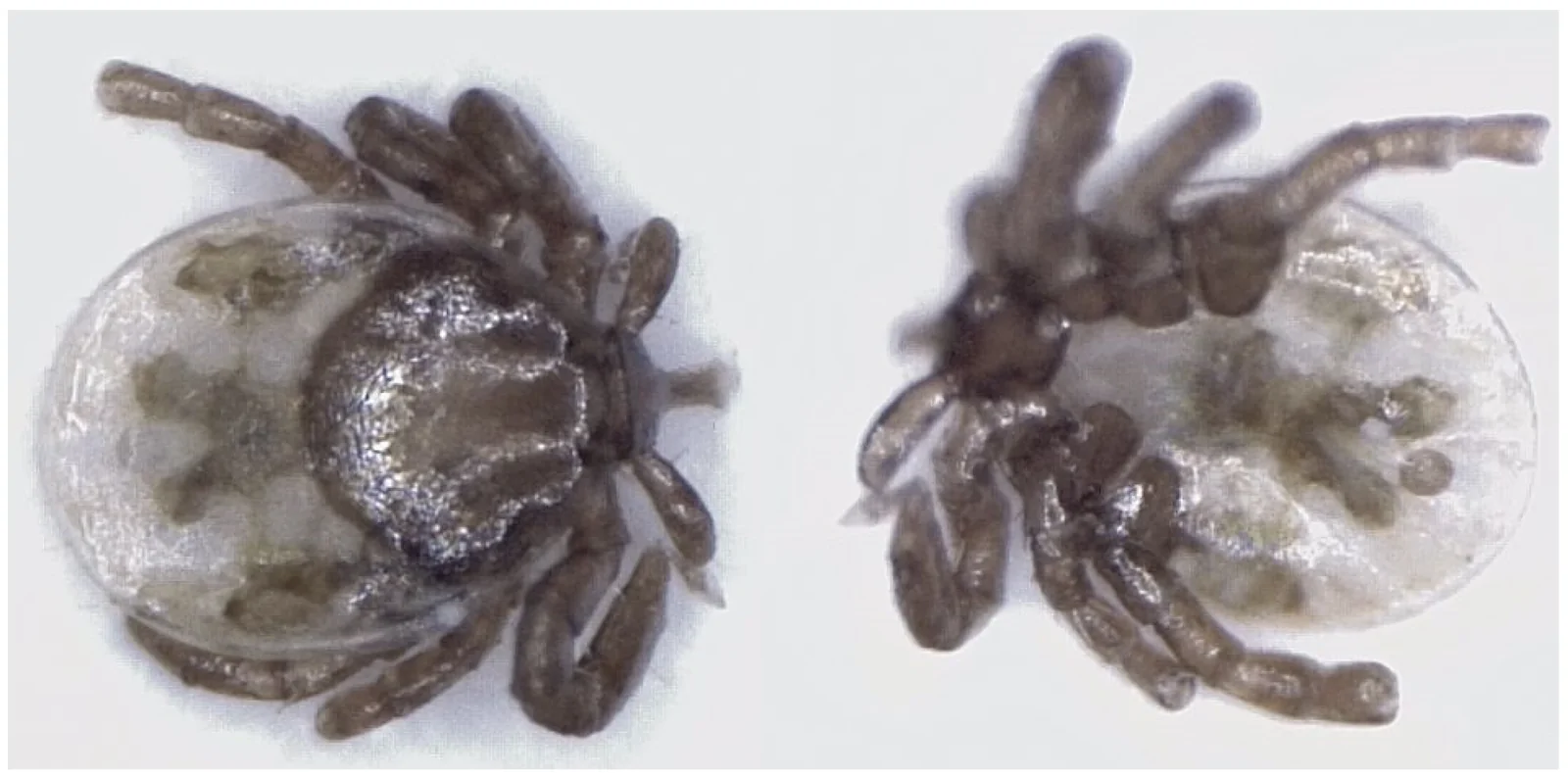
Photograph of a tick larva from the dorsal (**left**) and ventral (**right**) sides.

**Figure 2:**
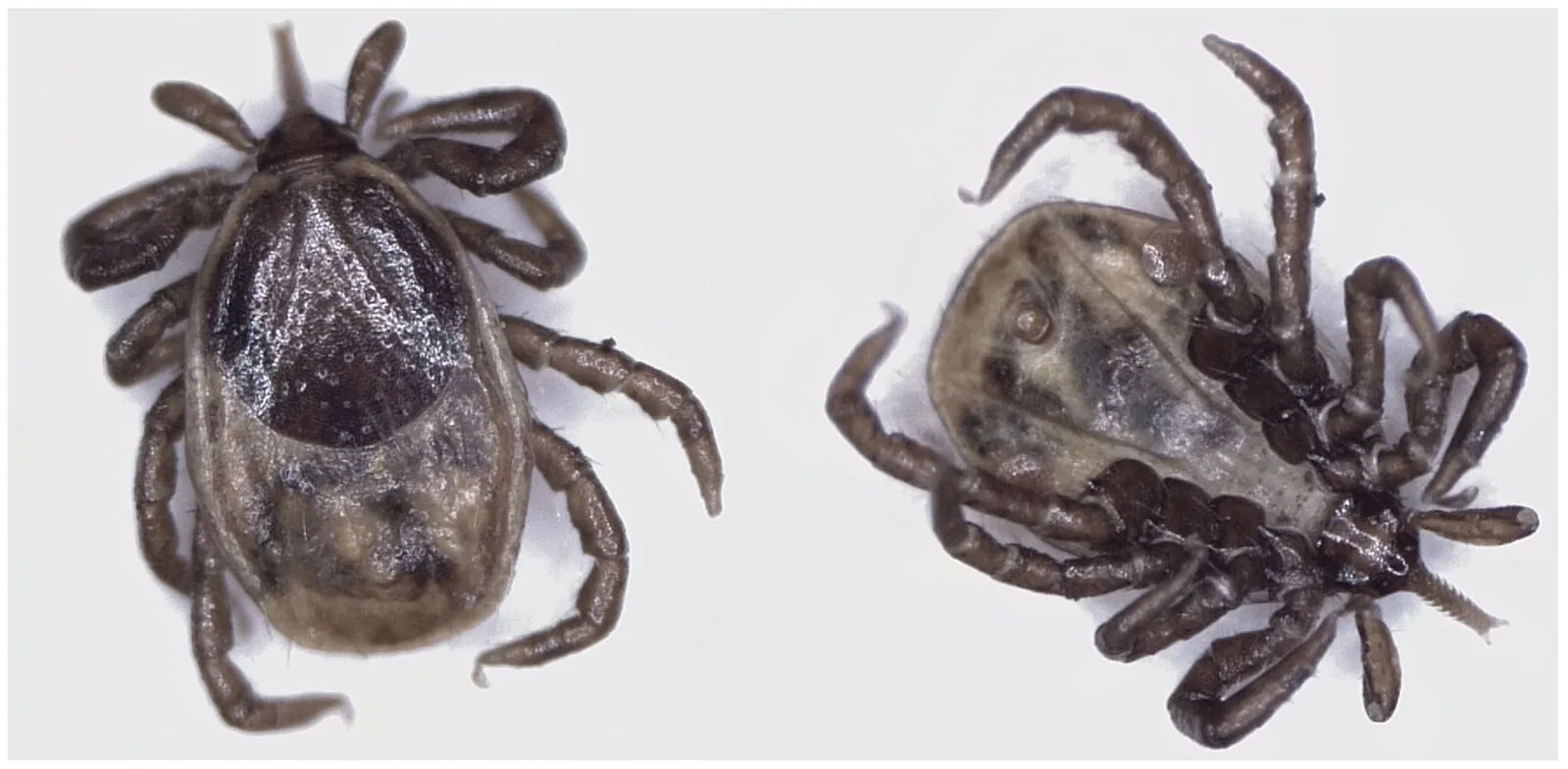
Photograph of a tick nymph from the dorsal (**left**) and ventral (**right**) sides.

**Figure 3:**
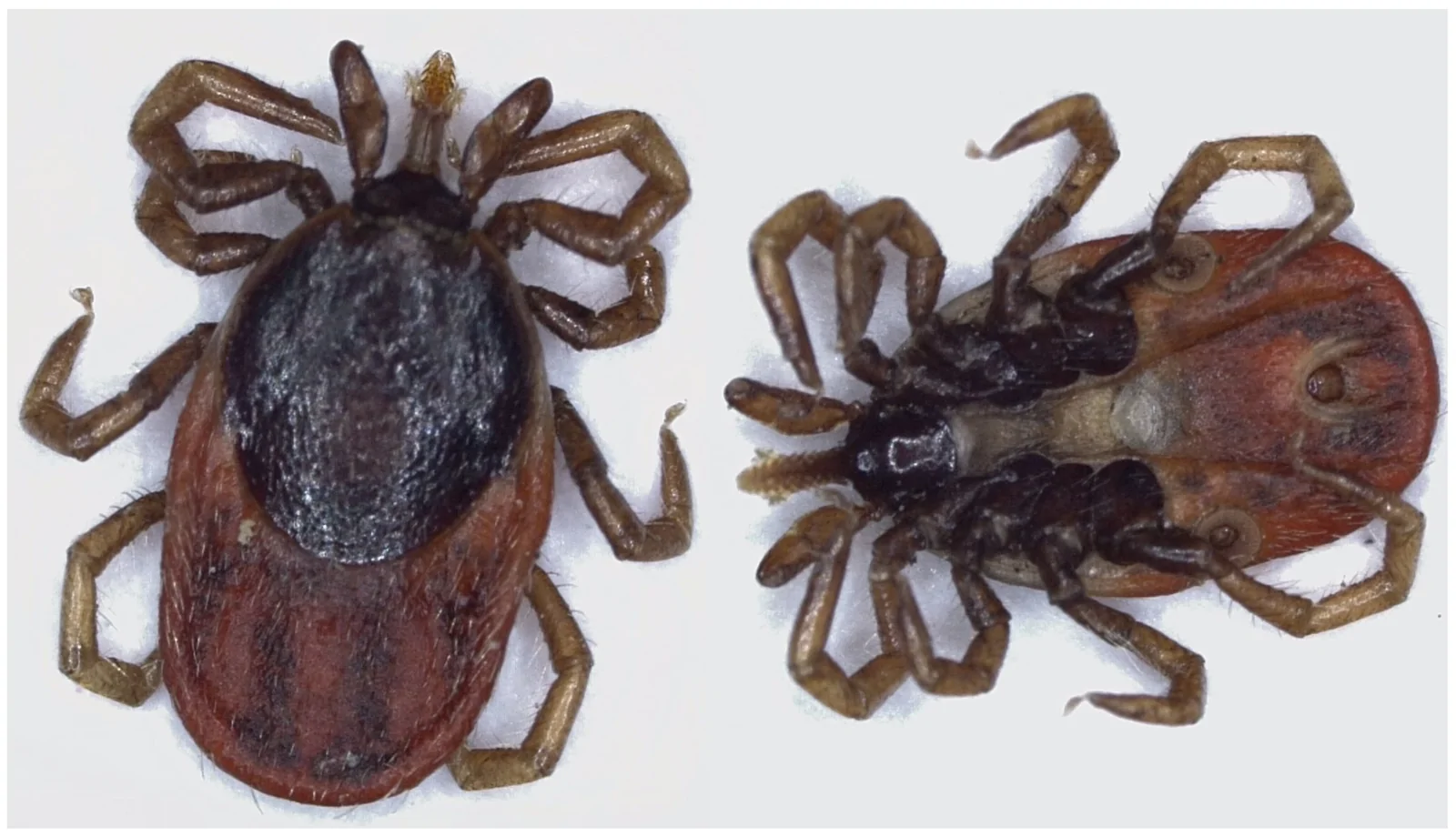
Photograph of a female adult tick from the dorsal (**left**) and ventral (**right**) sides.

**Figure 4:**
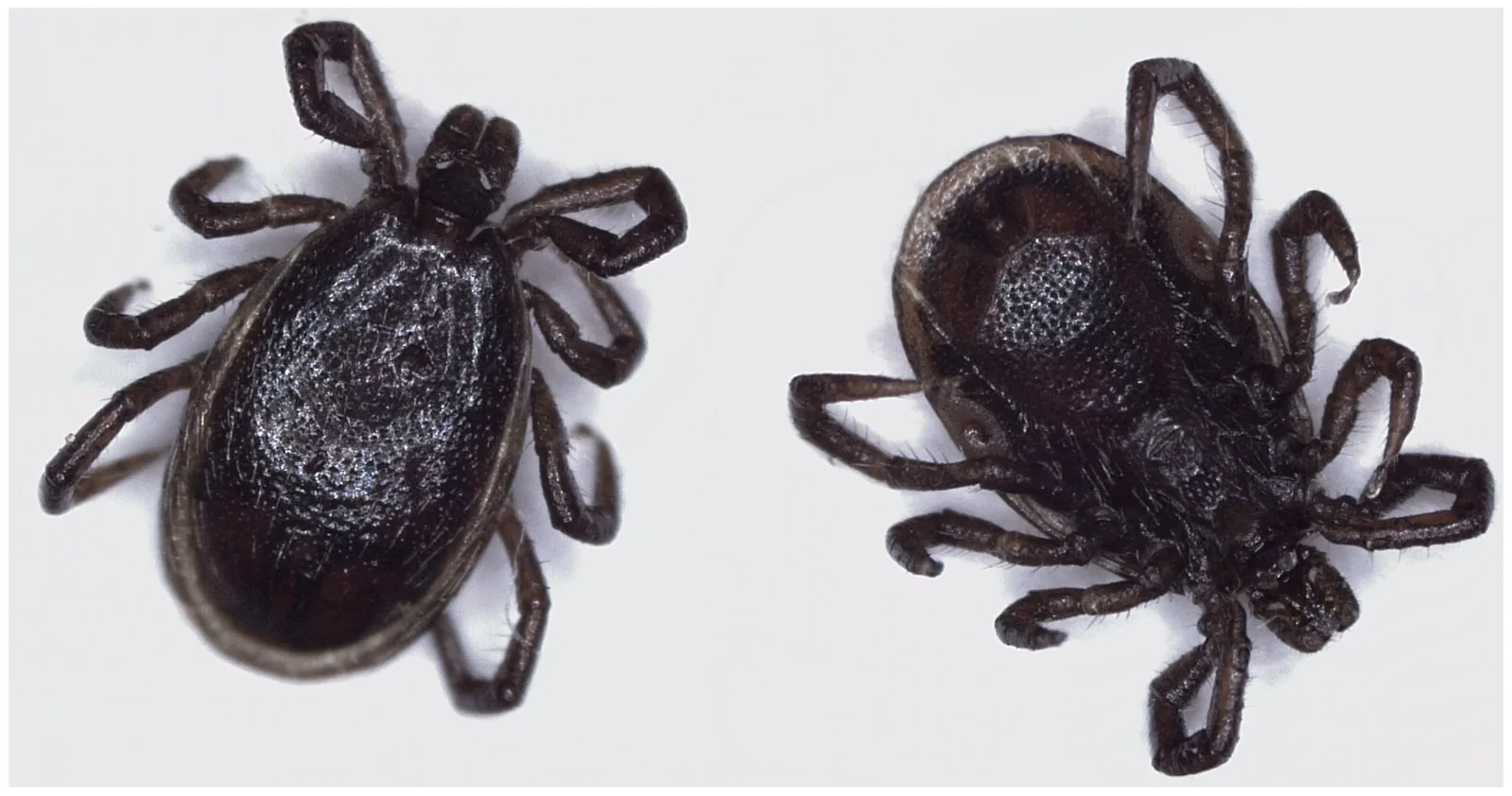
Photograph of a tick male adult from the dorsal (**left**) and ventral (**right**) sides.

*Ixodes ricinus*, the most common tick species in Central Europe [1,2], is an external parasite tick from the Ixodidae family that feeds on wild and domesticated animals, also targetting humans. This tick poses a significant biological threat, transmitting at least 20 pathogens of medical and veterinary importance [3], including the tick-borne encephalitis virus, *Borellia burgdorferi* spirochetes—the causative agents of Lyme disease (also known as *Lyme borreliosis*) [4], and *Anaplasma phagocytophilum*, the agent of human granulocytic anaplasmosis [5]. The most clinically relevant pathogens among them are the tick-borne encephalitis virus (TBEV) and *Borrelia burgdorferi* (TB). In Poland, *I. ricinus* is the predominant tick species and the primary vector of tick-borne diseases in humans [6]. *Ixodes* ticks typically complete their life cycle within 3–6 years, depending on environmental conditions [7]. After hatching, they pass through three active stages—larva, nymph, and adult—each requiring a blood meal from a host [8]. Each stage of the tick’s life cycle (larvae (Figure 1), nymphs (Figure 2), adults (Figure 3 and Figure 4)) associated with specific hosts is crucial for its survival, and the risk of pathogen transmission increases progressively throughout these stages. Immature stages feed on small mammals, birds, or reptiles, while adults primarily feed on large mammals [9].

Developmental timing is influenced by environmental factors such as host availability, species selection, and climate [10]. Feeding lasts 3–5 days for larvae, 4–7 days for nymphs, and 7–11 days for adult females [11]. Mating occurs during adult female feeding, and one female may lay 2000–2500 eggs, occasionally up to 4000 [12]. The extended life cycle is primarily due to the use of three hosts and the need for off-host moulting after each blood meal [7]. Ticks may also enter diapause in response to unfavorable conditions, which prolongs development [13]. Since adult ticks feed on the blood of at least three different hosts over their lifetime, the likelihood of pathogen transmission increases with each successive stage. Pathogens acquired from one host can be transmitted to new hosts during subsequent feedings [14]. Berglund and co-workers conducted a comprehensive epidemiological study on Lyme borreliosis (LB) in southern Sweden [15]. They found that neurological symptoms were significantly more common (20%) in LB patients who had been bitten by ticks on the head or neck, compared to those bitten on other parts of the body (7%). This suggests that the tick’s preferred attachment site on the human body could have clinical relevance [16].

According to a study conducted by Wilhelmsson and colleagues in Sweden between May 2008 and November 2009 on *Ixodes ricinus* ticks and their attachment sites (based on data from 1881 ticks), the legs were the most common attachment site in both men and women (51% of 597 ticks in men and 49% of 1051 ticks in women). This was followed by the trunk/back (20% and 24%, respectively) and the arms (19% and 17%, respectively). A significantly higher proportion of men (9%) than women (5%) reported ticks attached to the groin/genital area (*p* = 0.001). Conversely, a significantly higher proportion of women (5%) than men (1%) reported ticks attached to the head/neck region (*p* < 0.001). No other significant differences in tick attachment sites between men and women were observed [16]. Among the 1881 *I. ricinus* ticks examined, adult female ticks were significantly more frequently attached to the head/neck region (*p* < 0.001), the torso/dorsum (*p* < 0.001), and the groin/genital area (*p* < 0.01) compared to nymphs at the same body sites. In contrast, nymphs were significantly more often found on the arms (*p* < 0.001) and legs (*p* < 0.001) than adult female ticks. Due to the low number of adult male ticks and larvae, these stages were excluded from the statistical analyses [16].

The duration of tick feeding, including salivation and blood ingestion, is important for transmission of pathogens. The transmission of tick-borne pathogens often requires a period of reactivation and/or replication before they can be transferred via tick saliva to a naïve host [17]. For instance, effective pathogen transmission by ixodid ticks generally necessitates a minimum attachment duration of approximately 15 min for Powassan virus [17], 1 h for tick-borne encephalitis virus (TBEV) [18], and 3–24 h for *Ehrlichia* spp. [19], *Anaplasma* spp. [20], and *Rickettsia* spp. [20]. In the case of *Babesia* spp. [21] and *Borrelia* spp. [22,23], a longer feeding period of 24–48 h is typically required. Therefore, promptly locating and removing ticks from the skin is essential. Although TBEV virions may be transmitted within the first hour after tick attachment [16,18], early removal is strongly recommended, as the viral load in the tick’s salivary glands appears to increase with the duration of feeding. It is reasonable to assume that the longer a TBEV-infected tick remains attached, the greater the dose of virus transmitted to the host [16]. In contrast, *Borrelia burgdorferi* spirochaetes are not transmitted immediately, but the risk increases with the duration of tick feeding [24,25]. The risk may, in fact, already be present during the first 24 h of tick feeding [24]. Notably, Borrelia spirochetes have been detected in tick salivary glands and other tissues both prior to and at the onset of feeding [26]. All genospecies of *Borrelia burgdorferi* sensu lato, with the exception of *Borrelia afzelii*, have been detected in both the midgut and salivary glands of *Ixodes ricinus* ticks [22,27]. This suggests that transmission could occur during the early phase of feeding, before the engorgement stage begins [28]. In general, however, tick-bitten people that remove ticks later than 24 h of tick attachment are more likely to develop localised and systemic symptoms [29], probably due to injected tick salivary gland proteins and/or due to transmitted pathogens.

The prolonged time required for transmission of *Borrelia* spirochetes may be related to the metabolic activity of the tick vector, as described by Hoxmeier et al. [30]. *Borrelia burgdorferi* spirochetes lack the ability to perform de novo biosynthesis of nucleotides, amino acids, fatty acids, and enzyme cofactors. Consequently, they are entirely dependent on the blood meal not only for essential metabolic intermediates but also for environmental signals that initiate their migration within the tick [31,32]. Upon ingestion of blood, a metabolic cascade is activated in the tick midgut, which in turn influences the phenotype and activity of the colonizing spirochetes [33].

In unfed nymphs, nutrient-deprived *Borrelia* remain in a largely uncharacterized, metabolically quiescent state. During the first 24 h of nymphal feeding, the number of spirochetes in the tick gut remains low [34]; however, as feeding continues, their population expands exponentially [26], placing an increasing metabolic demand on the tick. As the spirochetes are exposed in the tick midgut to blood from the vertebrate host, they begin to replicate, penetrate the midgut epithelium, and migrate to the salivary glands, from which they are transmitted to the host during feeding.

Several studies have reported that larvae, nymphs, and adult ticks whose blood meal was interrupted can readily reattach to a new host and resume feeding [35,36,37]. This phenomenon, referred to as “interrupted feeding”, reflects the ability of partially fed ticks to survive, reattach to a new host, and reach full engorgement. Notably, this interrupted feeding pattern where ticks resume feeding on a second host within the same feeding cycle—has been shown to significantly reduce the time required for transmission of tick-borne pathogens such as *Borrelia burgdorferi*, *Rickettsia rickettsii*, and *Babesia canis* [22].

According to the study by Wilhelmsson et al. [16], 65% of the 1775 *Ixodes ricinus* ticks collected during the study were removed from the body more than 24 h after attachment. These ticks were submitted by 1770 participants. Older people, compared to younger ones, and men, compared to women, tended to detect ticks later, increasing the likelihood of prolonged feeding and, consequently, a higher risk of pathogen transmission. Most of the removed to late ticks were therefore nymphs, which are considered the most important life stage in the transmission of *Borrelia burgdorferi* sensu lato and TBEV to humans. This is due to their high abundance in nature and their small, inconspicuous appearance, making them more difficult to detect on the human body.

Although adult ticks particularly females are more likely to be infected with *Borrelia* [38], fewer were removed during the study. Moreover, their larger size and more visible appearance likely make them easier to notice and remove before pathogen transmission occurs. In this context, despite their higher infection rates, adult females may pose a relatively lower risk for Lyme borreliosis than nymphs. Notably, unfed larvae are rarely infected with either *Borrelia* [39] or TBEV [40].

**Figure 5:**
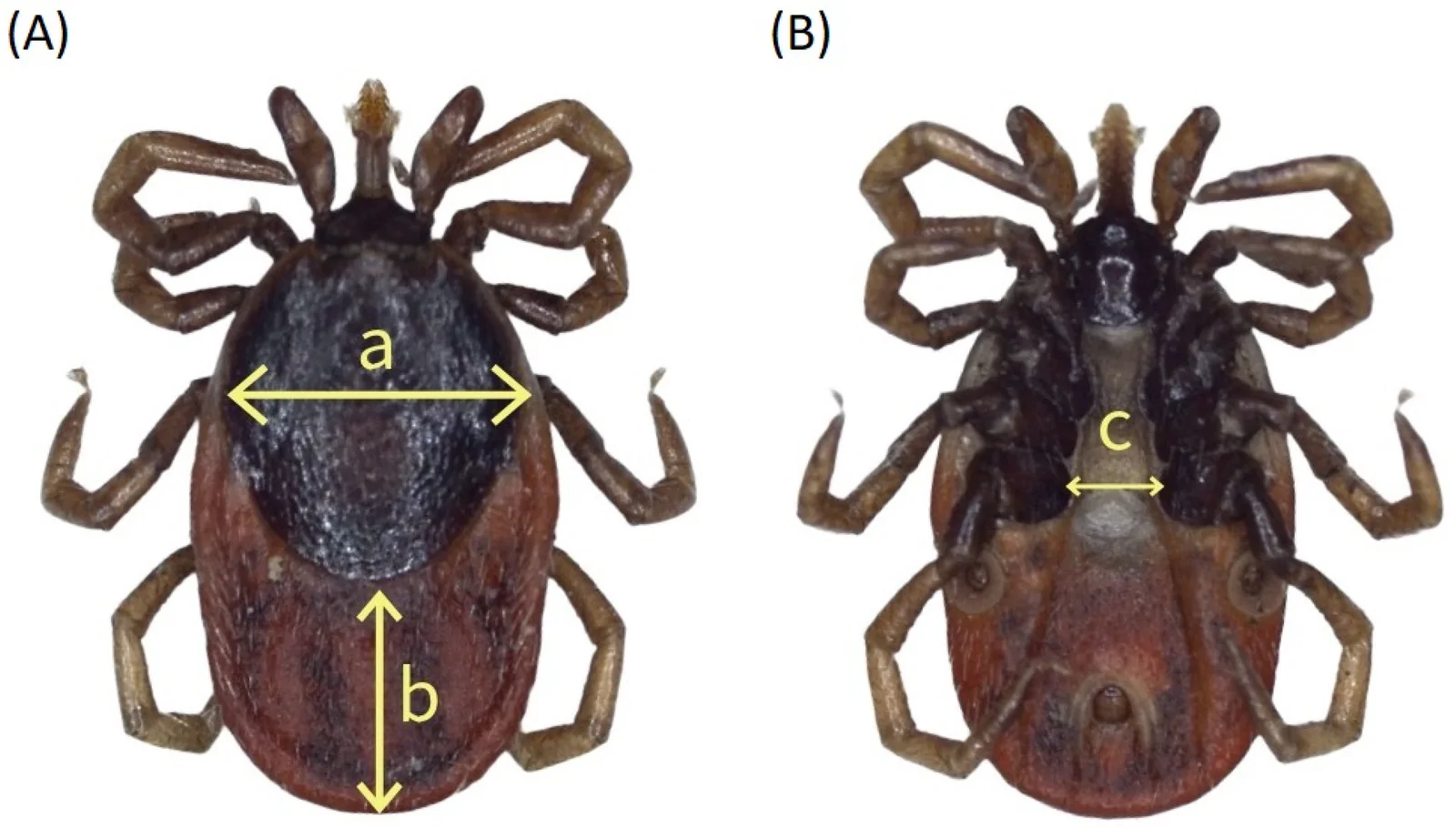
Dorsal (**A**) and ventral (**B**) views of an adult *Ixodes ricinus*. The scutal index is defined as the ratio between the length of the alloscutum (a) and the width of the scutum (b); the coxal index is defined as the ratio between the length of the alloscutum (a) and the distance between the basal coxae of the fourth pair of legs (c, coxal gap).

As demonstrated, the length of *Ixodes ricinus* tick feeding plays a crucial role in determining the risk of infection with diseases caused by the aforementioned tick-borne pathogens. Currently, studies aimed at determining tick feeding duration commonly rely on changes in tick body parameters, such as the scutal index (the ratio of idiosoma length to scutum width) or the coxal index (Figure 5) [16]. The method consists of the following steps: identification of the tick species, developmental stage, and sex based on microscopic images; measurement of the scutum width and the idiosoma length; and calculation of the scutal index (the ratio of the length of the idiosoma to the width of the scutum). In the case of the coxal index, the distance between the basal coxae of the fourth pair of legs is measured (across the ventral abdomen of the tick), and the index is calculated as the ratio of this distance to the width of the scutum [29,41]. The scutum width is constant for a given tick and does not change during feeding and engorgement [42]. Knowing the values of the indices, tick feeding duration can be estimated using the regression equations described by Gay et al. [29], which differ depending on the developmental stage and sex of the tick. As demonstrated by the study conducted by Gay et al. [29], the values of the scutal index and coxal index remain unchanged during the first 12 h of tick feeding. This observation is related to the multistage nature of tick feeding, in which the body mass of the attached tick does not increase significantly—if at all—during the initial (preparatory) phase, which lasts approximately 24 h. The majority of the blood meal is ingested during the final 12–36 h of feeding, known as the rapid engorgement phase [2]. For both nymphs and adult females of *Ixodes ricinus*, measurements of the scutal index indicated slower changes in tick dimensions during the early phase of feeding compared to the coxal index. Therefore, the coxal index may be considered a more accurate measure of feeding progression during the first 24 h of attachment. However, in the later stages of feeding, the coxal index becomes progressively less sensitive and more variable than the scutal index [2].

As demonstrated by Meiners et al. [41], the scutal index, as a direct measure of tick engorgement, may serve as an indirect indicator of *Borrelia* transmission risk, given its correlation with feeding duration. The risk of host infection might be distinctly higher when a *Borrelia burgdorferi*-infected *Ixodes ricinus* nymph has a scutal index between 1.1 and 1.5, corresponding to approximately 36 h (~24–~40 h) of feeding. The infection risk may be very high if the detached tick has a scutal index greater than 1.5, indicating that it might have fed for more than 36 h.

According to a study conducted by Marina Žekić et al. [43] in northern Serbia between 2019 and 2023, patients presented to medical facilities with attached ticks. A total of 308 *Ixodes ricinus* ticks were removed from people, of which 111 (36.04%) were nymphs and 197 (63.96%) were adult females, while no larvae were detected. *Borrelia* spp. was identified in 56 ticks (17.8%) using real-time PCR. The prevalence of infection among ticks varied between 5.5% and 32.26% across the successive years of the study. When adjusted for the relative abundance of each developmental stage, the proportion of infected nymphs was comparable to that of adult females—or in some cases, even higher.

As demonstrated, the developmental stage, as well as the length of *Ixodes ricinus* tick feeding plays a crucial role in determining the risk of infection with diseases caused by the aforementioned tick-borne pathogens.

### Ticks Classification

Accurate identification of the developmental stage of ticks is a key component in scientific research involving these arthropods. Determining the developmental stage of ticks is essential in various types of research, including statistical studies involving both unfed and fed ticks, in vivo and in vitro laboratory experiments, epidemiological surveys assessing tick prevalence and infection rates in specific regions, as well as clinical studies based on ticks collected from individuals seeking medical attention. Traditional methods of tick identification rely on visual, primarily microscopic, examination of their morphological features, such as the presence, shape, and coloration of a dorsal shield scutum, the length, shape, and orientation of the mouthparts, the number, shape, and segments of legs. Identification keys [1,6] allow researchers to systematically compare observed tick traits with documented characteristics, using a sequence of descriptive questions to narrow down possible species matches.

Each developmental stage exhibits distinct morphological features, making it essential to consider the tick’s life stage during identification. Larvae, for instance, possess three pairs of legs, while nymphs and adults have four. Sexual dimorphism is clearly evident in adults: adult male ticks are characterized by a dorsal scutum that covers nearly the entire body surface in males, while in females (but also in nymphs) it covers only part of the body. Additionally, females usually have distinct grooves on the dorsal shield and measure between 3.0 to 3.6 mm in length, whereas males are smaller (2.4 to 2.8 mm), with relatively small chelicerae [6].

Although traditional identification techniques are time-consuming and require specialized entomological expertise, they remain fundamental to both entomological and epidemiological research. Convolutional neural networks (CNN) have revolutionized species identification by applying advanced AI algorithms to accurately identify and classify various biological taxa, such as bacteria, viruses, plants, and animals [44]. Diverse types of data, such as macro- and microscopic images, genomic and proteomic data, sound recordings, and behavioral observations can be utilized in training neural networks. These approaches are particularly valuable in the medical field, as they assist in identifying organisms harmful to human health, like bacteria, parasites, and disease vectors such as insects, facilitating improved infectious disease management. Recently, advanced deep learning approaches for recognizing species images have been implemented in the management of infectious diseases transmitted by insects, like ticks or mosquitos [45,46,47]. Compared to molecular techniques like genotyping, image-based computer vision methods are non-invasive and non destructive, which is especially beneficial when working with small organisms that may otherwise be destroyed during analysis. In this study, we developed a CNN-based computer vision approach for rapid, automated indentification of *Ixodes ricinus* life stages. This solution may be particularly beneficial for untrained healthcare workers lacking taxonomic expertise, as well as entomologists who can use it to complement traditional methods, improving efficiency in research and monitoring. In parallel, we developed an application based on neural networks to determine the developmental stage of ticks, with the aim of further automating future tick-related research. This program is intended to accelerate work in upcoming studies. As part of our research, we also set out to create a large database of images representing various developmental stages of ticks, with an emphasis on highlighting their morphological differences.

## 2. Dataset Description

**Figure 6:**
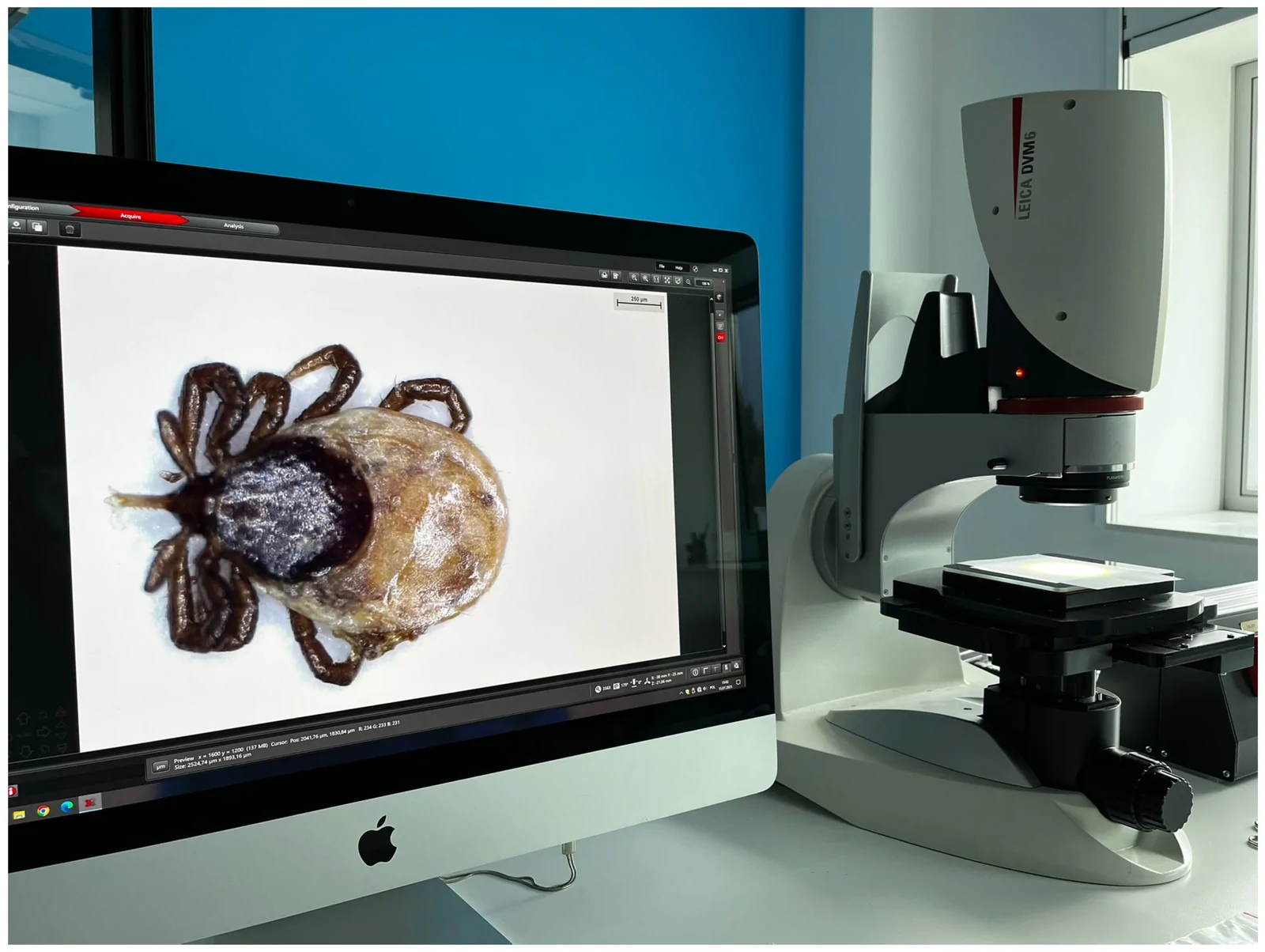
DVM6A microscope equipped with the LAS X system (Leica Microsystems Heidelberg GmbH).

The dataset consisted of microscopic images of ticks collected from green areas in Zabrze, Poland, between April and October 2022. Ticks were gathered using a drag cloth method [1,48]. A total of 564 tick specimens were collected, preserved at −80 °C and photo-documented using DVM6A digital microscope (Leica Microsystems Heidelberg GmbH, Wetzlar, Germany). Morphological analysis was conducted by capturing high-resolution images of ticks using a Leica DVM6A (Figure 6) digital microscope equipped with a motorized stage and a 16:1 zoom system, offering a magnification range from 10× to 2350× and capable of resolving structural details as small as 0.4 µm. The system features a high resolution 1/2.3” CMOS camera sensor with a native resolution of 3648 × 2736 pixels and a pixel size of 1.67 × 1.67 µm. Images were acquired in high-resolution format (1600 × 1200 pixels, RGB, saved as JPG and TIFF files), showing dorsal and ventral views of the ticks. During image acquisition, the LED illumination ring of the DVM6A microscope was configured using the “Scene” mode in the LAS X (v5.2.2) software, which allows precise control over both the number and angle of active light sources. This enabled us to adjust the direction of illumination to minimize shadowing and eliminate surface reflections especially on the tick’s sclerotized dorsal shield. Light intensity was manually optimized for each image to ensure maximum anatomical detail visibility while avoiding overexposure (“burnout”) of bright regions. These adjustments ensured uniform and high-quality illumination across all specimens, improving both visual interpretation and the robustness of feature extraction in the CNN model.

CMOS, which stands for Complementary Metal Oxide Semiconductor, refers to a type of imaging sensor widely used in modern digital microscopy and photography. The first CMOS imaging sensor specifically, an active pixel sensor was developed in 1992 at NASA’s Jet Propulsion Laboratory (USA) [49]. Although early versions exhibited limited light sensitivity and a poor signal-to-noise ratio, they offered several advantages over traditional CCDs (Charge-Coupled Devices), including lower power consumption, higher integration potential, a more compact form factor, and reduced manufacturing costs [50]. The subsequent development of CMOS technology was largely driven by the growing demand in the mobile phone camera market. Over time, significant improvements were achieved in key performance parameters such as quantum efficiency, dynamic range, and signal to noise ratio. As a result, the image quality produced by CMOS sensors has become comparable to or even superior to that of CCDs in many applications [50].

CMOS image sensors exhibit a range of advantages that make them highly attractive for various imaging applications. One of their most prominent benefits is low power consumption, which is particularly advantageous for portable and energy efficient systems [51]. Their random access readout architecture enables high-speed image acquisition and region-of-interest (ROI) functionality, allowing selective data capture and faster processing. A major strength of CMOS technology lies in its compatibility with standard semiconductor fabrication processes, which allows for seamless integration of analog and digital circuits such as signal processing units and analog to digital converters directly on the sensor chip. This high level of integration contributes to smaller system size, reduced cost, and enhanced design flexibility, making CMOS sensors ideal for high-resolution, real-time, and multifunctional imaging applications.

However, CMOS sensors are not without limitations. A key disadvantage is their higher dark current, which can degrade image quality, particularly under low-light conditions. Additionally, due to the presence of multiple active components within each pixel, CMOS sensors are more prone to temporal noise, including shot noise, reset noise, and thermal or flicker noise. Another common issue is fixed-pattern noise (FPN), which results from spatial non-uniformities across the sensor array due to process variations and device mismatches. In particular, column-level FPN, arising from inconsistencies in column amplifiers, may lead to visible artifacts such as banding or streaks in the image. Furthermore, pixel-to-pixel non-uniformity may necessitate additional calibration or correction algorithms to ensure consistent output quality [51].

The dataset consisting of 1323 tick images was prepared for model training by applying several preprocessing steps. All specimens were identified morphologically as *Ixodes ricinus* by standard taxonomic keys [1,6] and categorized by life stages: larvae (56 images), nymphs (1129 images), adult females (58 images), and adult males (80 images). Preprocessing included resizing, normalization, and augmentation to ensure the dataset is suitable for training the model.

Data augmentation was a key step that improved the model’s performance and generalization. The augmentation included: image rotations (up to 90 degrees), zooming (by 10%), horizontal and vertical shifts (up to 10%), flipping, and shear transformation (up to 10 degrees). These image transformations were selected to simulate realistic variations in tick positioning, orientation, and imaging conditions that may occur during manual photodocumentation. Importantly, all transformations were applied with constraints to preserve the morphological features crucial for developmental stage classification (e.g., body proportions, leg visibility, and gnathosoma orientation). No transformations that could introduce artificial artifacts or obscure key anatomical structures (e.g., strong blurring, cropping or excessive changes in brightness or saturation) were used. This strategy effectively increased the diversity of training examples and reduced overfitting. The images were resized from 1600 × 1200 to 320 × 320 pixels, and pixel values were normalized to the [0, 1] range. After preprocessing, the dataset was split into training and validation sets.

## 3. Methods

The data was divided into a training set (200 images per class) and a validation set (50 images per class) with equal class representation ensured through augmentation. Validation was performed on non-augmented data, allowing for a reliable quality assessment. The learning process was monitored using ReduceLROnPlateau and EarlyStopping, which prevented overfitting.

The software was implemented entirely in the Python programming language [52,53], and the TensorFlow library (v2.11) was used to create the models used in the experiments [54].

### 3.1. Training on the Pre-Trained Xception Model

The CNN model was built using the Xception architecture as a base, with additional layers such as Flatten, BatchNormalization, Dense, and Dropout. CNN was pre-trained on the ImageNet dataset, enabling quick tick classification adaptation through transfer learning. The Xception network acted as a feature extractor, with additional dense layers added to classify images into four categories: larvae, nymphs, adult males, and adult females. This allowed the model to effectively recognize patterns characteristic of different tick developmental stages, significantly improving its accuracy.

### 3.2. Training

The model was trained for 30 epochs using the Adam optimizer, which automatically adjusted the learning rate for efficient convergence. The categorical cross-entropy loss function measured the difference between predictions and actual class labels, guiding the training process to enhance the model’s image classification accuracy.

### 3.3. Evaluation

The model’s performance was evaluated over 30 epochs in both the training and validation sets. Training was further optimized with callbacks like ReduceLROnPlateau and EarlyStopping, which adjusted the learning rate and stopped training when no further improvements were detected.

### 3.4. Model Explainability: Grad-CAM Method

To analyze the CNN model’s decision-making process in classifying the development stage of *I. ricinus*, the Grad-CAM (Gradient-weighted Class Activation Mapping) [55] technique was used. Grad-CAM generates activation maps that highlight which areas of an image most influenced the model’s classification. These maps are based on the gradients of the loss function concerning the activations of the model’s convolutional layers, with heatmaps showing the intensity of influence—areas with higher intensity had a greater impact on the model’s decisions.

The Grad-CAM is one of the key explainable artificial intelligence techniques used in the field of image classification. Explainable AI (XAI) is concerned with creating machine learning models whose decisions are understandable to humans. It encompasses a variety of techniques, which makes it applicable to various fields of artificial intelligence and machine learning, including computer vision and image analysis [56]. It allows us to not only get results, but also, to some extent, to understand why the model made a certain decision.

Our goal is to create a system for specialists (scientists, researchers), which is why we focus on creating a database of high-quality images obtained by a microscope equipped with CMOS (Complementary Metal–Oxide–Semiconductor) sensors. One of the most important elements at this stage of the project is to ensure the explainability of our model.

Accurate photos are necessary for at least two reasons. Firstly, to create a CNN-based model to recognize the developmental stage of a tick, and secondly, to read certain anatomical dimensions from an image.

To verify the length of feeding time, various measures are used, requiring precise dimensions of the corresponding anatomical details (body parts) of the tick [41]. For example, depending on the method used, these will be the dimensions of the scutellum, the length of the abdomen, or the distance between the last pair of legs. In principle, all methods require very detailed photos from different sides and the ability to read the dimensions, thanks to the knowledge of the exact scale of the photo taken.

Due to the fact that the creation of the database is spread over time (the difficulty of obtaining a large number of photos during one season), we have to rely on the available number of photos, fewer than we would like. Therefore, it is very important from our point of view to be able to quickly verify whether the appropriate details in the photo are taken into account when training our models (CNN network).

In our experiments, we use the Grad-CAM method [55]. Grad-CAM is a technique for explaining CNN decisions that visualizes which areas of the image were most important to the network when making a specific prediction. The Grad-CAM (Gradient-weighted Class Activation Mapping) method allows us to understand the operation of our models and better control the learning process. With Grad-CAM, we can actually see what parts of an image our network focuses on. This helps us confirm it is paying attention to the right visual patterns. It provides us with some degree of explainability that we need. The Grad-CAM method does not minimize the effects of learning on a smaller image database and does not prevent overfitting, but it does allow you to detect problems during training. If a model has been retrained on a small dataset, it often starts to “look” at specific, irrelevant details in training images, rather than learning general, relevant patterns. Using Grad-CAM, we can visually identify if the model is looking at the wrong places instead of the key features of the object. If Grad-CAM heatmaps show that the model is focusing on irrelevant regions for a given class, it is a strong signal that the model may be overtrained and is not generalizing well. Grad-CAM can help reveal that the model has learned to rely on unwanted correlations in the training data—it helps detect dataset bias.

Recognizing the stage of tick development through the CNN is an important element of our target system, but not the only one. It is the first stage of data processing. The next stages will include segmentation of the appropriate anatomical elements of the tick (such as the scutellum or legs), and then measuring the necessary sizes of the segmented elements. These stages will use software for classic computer vision methods (implemented, for example, in OpenCV [57,58]).

## 4. Results

**Figure 7:**
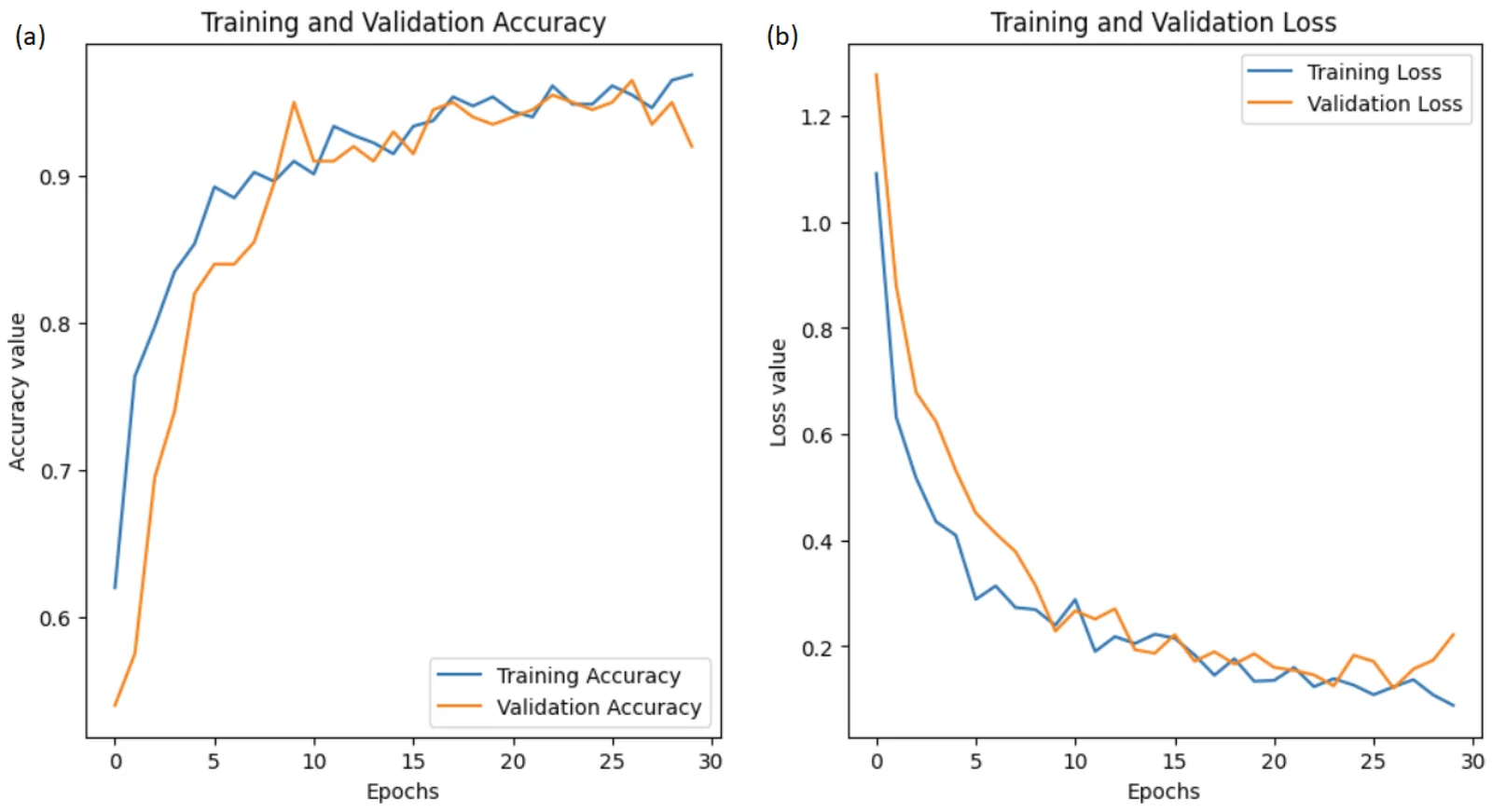
Results of classification: (**a**) training accuracy curve, (**b**) training loss curve. Accuracy and loss functions over 30 epochs during the training phase, RGB 320 × 320 pixels. Final accuracy: 98%.

The training and validation plots (Figure 7) show changes in accuracy and loss functions for training and validation RGB image sets over 30 epochs, with an initial accuracy of 95% and an improvement to 98% after fine-tuning the model.

When higher-resolution images (512 × 512 pixels) were used in subsequent tests, the accuracy increased to 98%, indicating that higher image resolution provides more significant information for classification.

**Figure 8:**
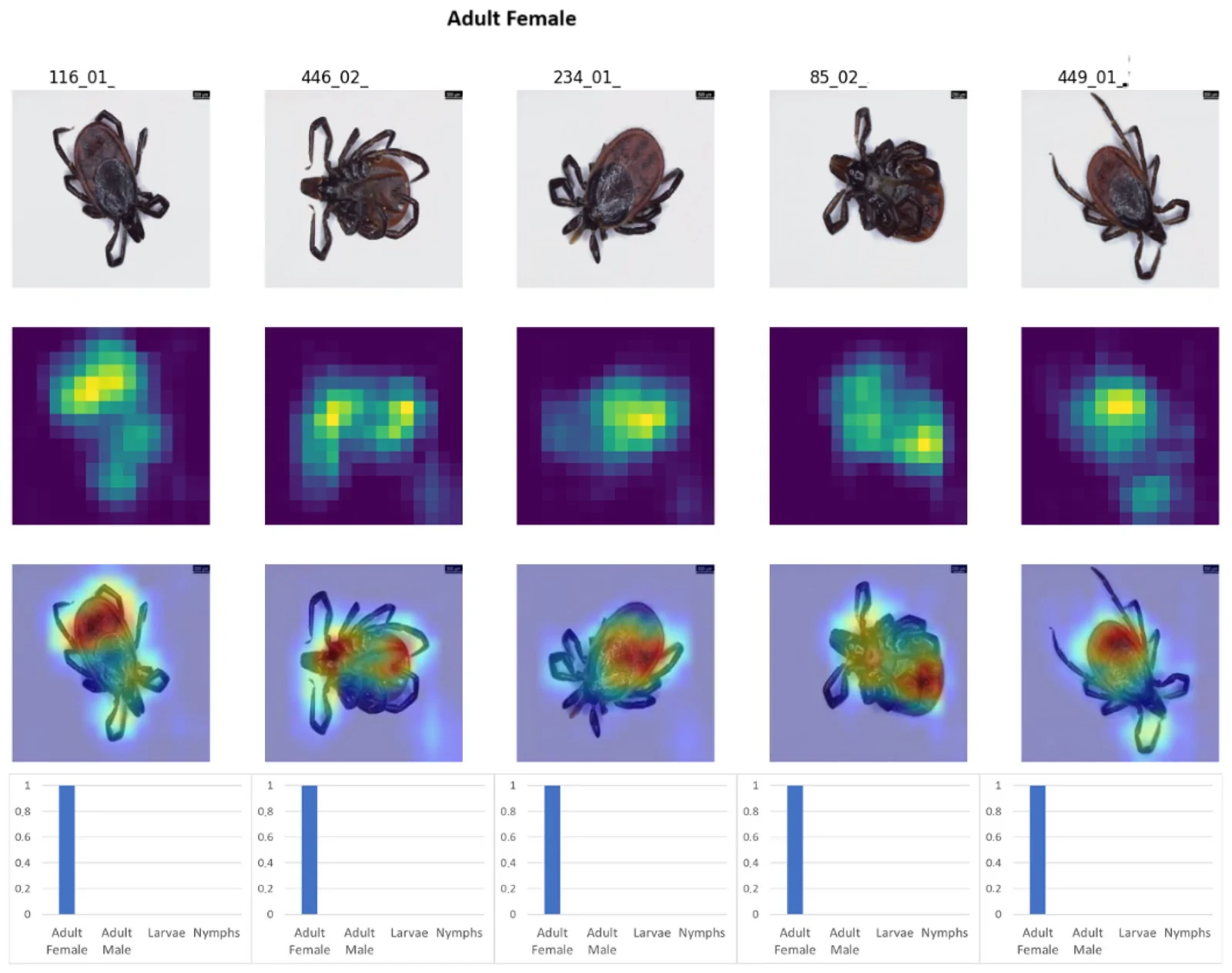
Grad-CAM visualizations showing areas of activation for adult female classification.

**Figure 9:**
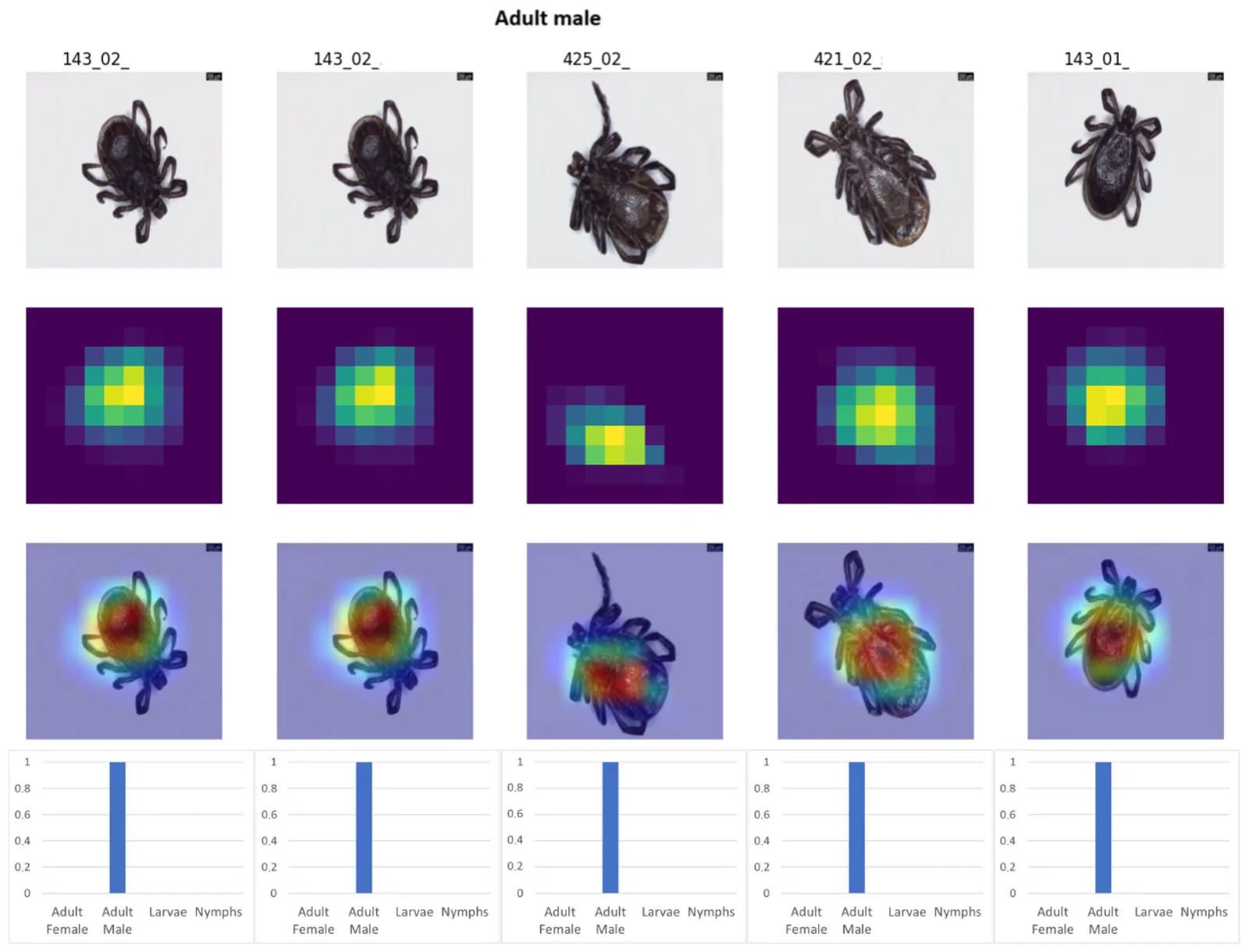
Grad-CAM visualizations showing areas of activation for adult male ticks.

The key features for tick classification, such as the dorsal shield of adult ticks, were highlighted by Grad-CAM visualizations (Figure 8 and Figure 9). These maps made it easier to interpret the model’s decisions by revealing the regions of the image that contributed the most to classification. For example, the model often focused on the central and peripheral areas of the tick’s body, suggesting these regions contained key distinguishing features that may not be visible to the naked eye.

Further insights were provided by bar charts that presented the model’s confidence levels for each classification. As shown in Figure 8 and Figure 9, the model displayed high confidence in classifying most images, particularly in distinguishing adult developmental stages, likely due to the distinct dorsal shield seen in adult specimens. Examples of visualizations and classification results for adult ticks were presented, providing a clear assessment of the model’s ability to identify these developmental stages.

### Impact of Grayscale and Testing on Unseen Images

**Figure 10:**
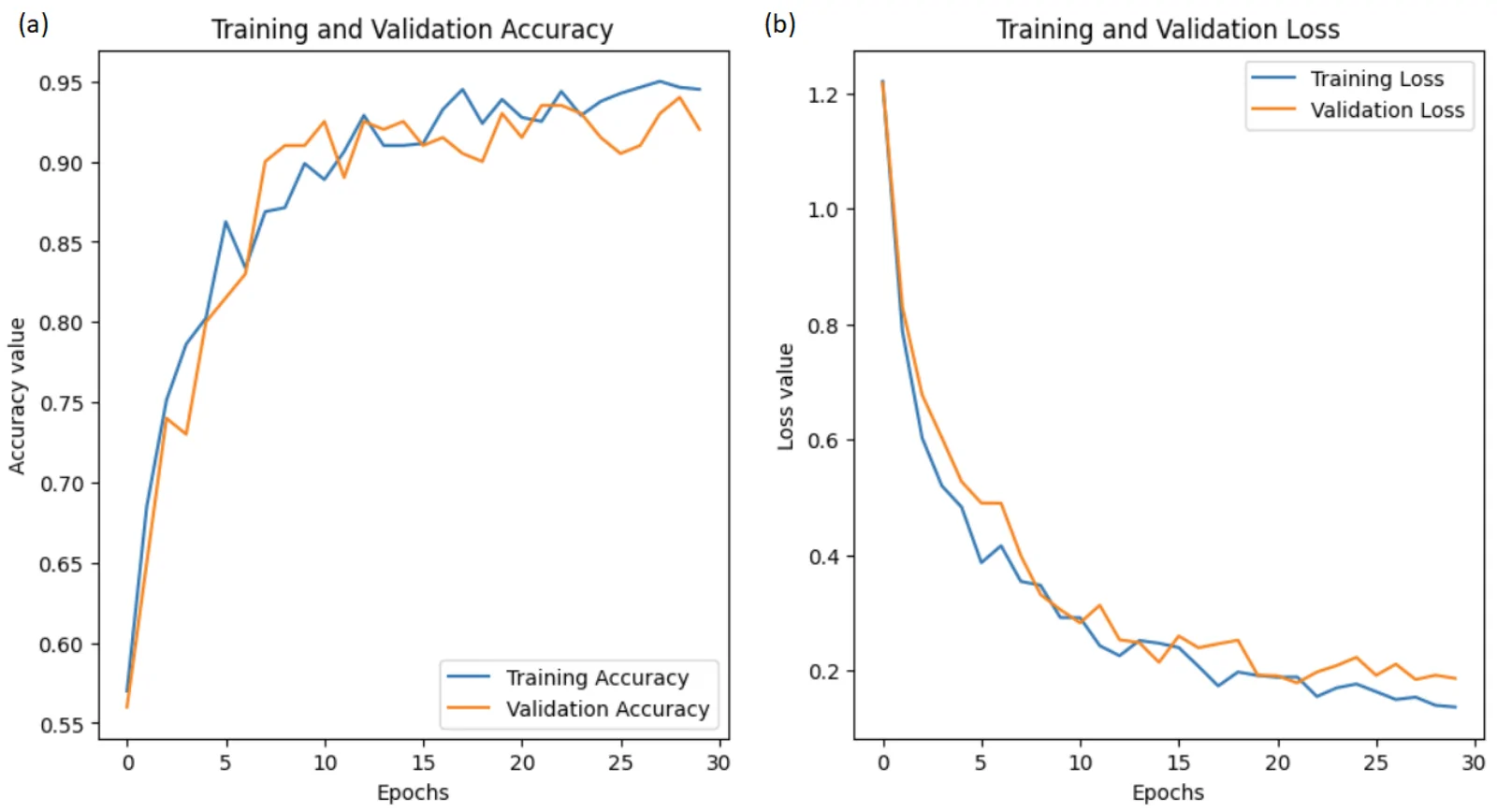
Results of classification on grayscale images: (**a**) training accuracy curve, (**b**) training loss curve. Accuracy and loss over 30 epochs, grayscale 320 × 320 pixels. Final accuracy: 95%.

The transition from RGB images (with three color channels: red, green, and blue) to grayscale images, which have only one channel, reduced the number of features the model needed to process. Despite this change, the model’s accuracy remained at 95%, indicating that it focused on similar regions of the image in both cases (Figure 10). This suggests that the model consistently identified the same features, regardless of whether the image was in color or grayscale.

The model was also tested on previously unseen images downloaded from the internet, featuring dorsal sides of ticks with identified developmental stages. Results showed that the model performed better on images with a uniform background, where the ticks were clearly visible and close to the camera. Challenges arose on images with more complex backgrounds or when ticks were farther from the camera. To improve the model’s performance, increasing the diversity of the training data by including more complex backgrounds and various distances could be beneficial. It is also possible to immerse ticks in boiling water and then transfer them to ethyl alcohol. These conditions lead to the rapid death of the ticks and the straightening of their legs, which would enhance the accuracy of specimen identification [6].

The model, tested on previously unseen microscopic images of ticks extracted from the human body, achieved an accuracy of 75%. This result suggests that the next step in model improvement should involve training with a larger dataset, including images of engorged ticks. In the case of engorged ticks, females significantly increase in size due to the absence of an exoskeleton covering the entire body. Similarly, engorged nymphs show notably enlarged abdomen and exhibit different idiosoma coloration from adults. For the classification of engorged individuals, the size of the abdomen and the morphology of the mouthparts may play a crucial role.

The CNN model demonstrated high accuracy in classifying the developmental stages of *Ixodes ricinus* ticks. During training on RGB images, the model achieved 95% accuracy after 30 epochs. Increasing the image resolution from 320 × 320 to 512 × 512 pixels improves the accuracy to 98%, confirming the importance of higher image resolution for precise classification (Figure 7).

Further tests on grayscale images showed that the model maintained a similar accuracy (95%), suggesting that the network effectively identified key morphological features, such as shape and structure, without relying on color information (Figure 10). The Grad-CAM was used to analyze which image regions were most critical for classification, revealing that areas like the dorsal shield of adult ticks were the key. These visualizations helped interpret the model’s decisions and identify potential classification errors (Figure 8 and Figure 9).

**Figure 11:**
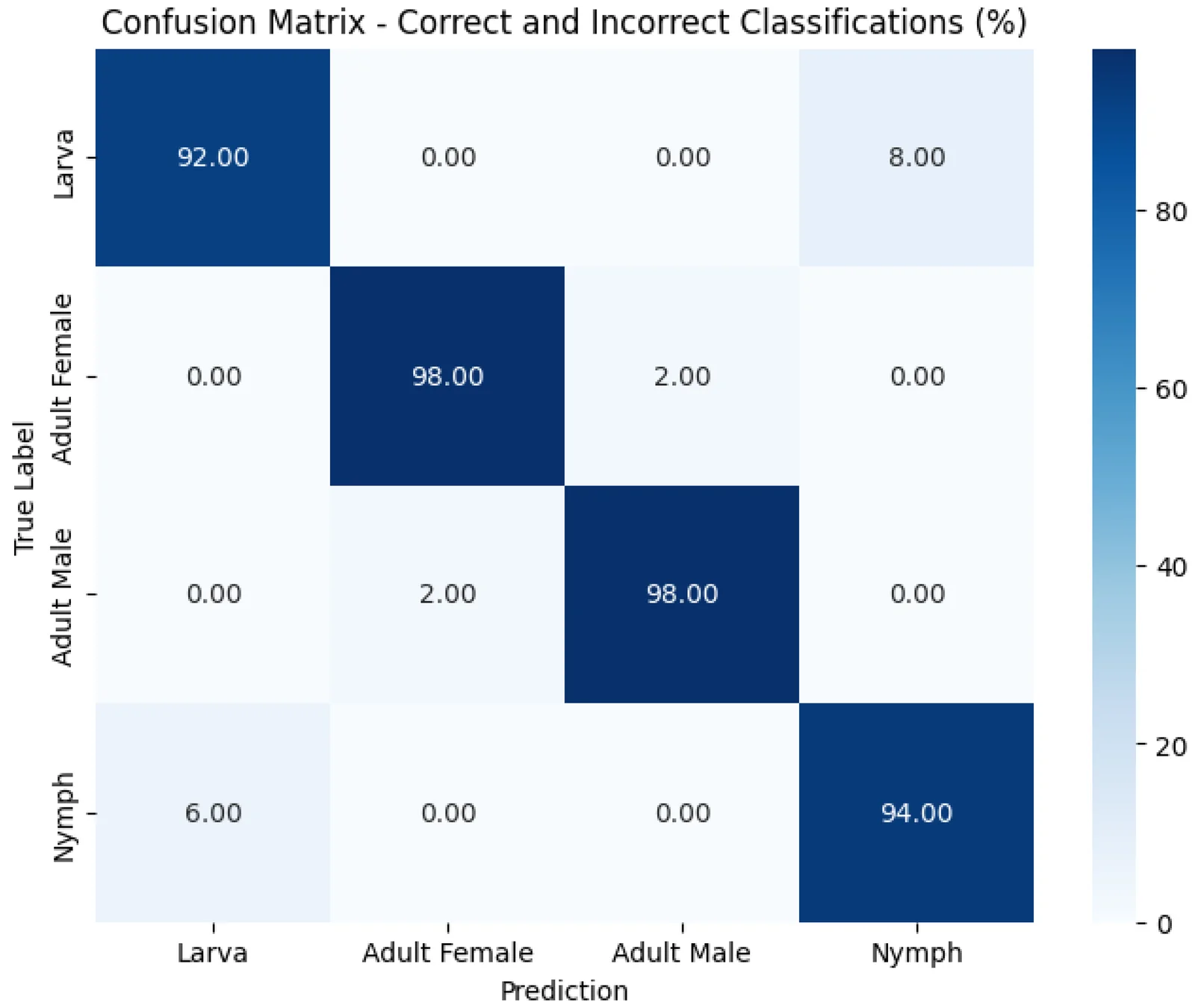
Confusion Matrix showing correct and incorrect classifications in percentages for each category.

Overall, the model exhibited high effectiveness and stability, achieving satisfactory results on both RGB and grayscale images. The confusion matrix (Figure 11) demonstrates a high classification accuracy, as evident from the high percentages along the diagonal (from the top-left to the bottom-right). The values on the diagonal represent the correct classification for each class. The main misclassifications occur between “Larva” and “Nymph” as well as between “Adult Female” and “Adult Male”. This could indicate similarities in features between these categories, which makes distinguishing them more challenging (Figure 11).

## 5. Discussion

Accurate identification of tick specimens and their developmental stages is essential in entomological studies as well as in the management of tick-borne diseases. Our developed solution for recognizing the developmental stages of the *Ixodes ricinus* introduces several innovative features. To the best of our knowledge, this is the first study utilizing the CNN model for the identification of European tick fauna. While convolutional neural networks have previously been applied to tick classification [46,59,60,61,62] those efforts primarily focused on species prevalent in North America, such as *Amblyomma americanum*, *Dermacentor variabilis*, *Ixodes scapularis* (Table 1). In contrast, our study was designed to address the need for an automated tool tailored to species commonly encountered in Central Europe, particularly *I. ricinus* and *Dermacentor reticulatus*. Due to the fact that all the specimens collected in our dataset were classified as *Ixodes ricinus* our research focused on distinguishing its developmental stages.

It is well known that the risk of pathogen transmission depends on the tick species. However, the developmental stage of the tick also plays a significant role in transmission risk. Studies indicate that particularly nymphs of *Ixodes ricinus*, and *Ixodes scapularis* are effective vectors of pathogens in humans due to their abundance in the environment and small size, which allows them to feed on hosts without being easily detected [63,64,65]. A tool for identifying the developmental stages of ticks has not yet been published. However, there is a pressing need for such solutions in agricultural studies, where real-time, in situ, monitoring of insect population based on the YOLO model provides crucial insight into the effectiveness of insecticides [66] or monitor population dynamics of endangered, ecologically important species such as bees [67].

Despite the relatively small dataset, the use of a uniform white background in the photodocumentation process and the high, microscopic quality of captured images improves our training process. Analyzing results from commonly used model architectures in ticks identification studies (Table 2), InceptionV3 and ResNet50, the highest accuracy was achieved with large, well-prepared datasets (e.g., 12,000 microscopic images with a high resolution of 3648 × 2736 pixels), where validation accuracy reached up to 99.50% [59]. Smaller datasets, such as those with 2130 images [60] or lower quality, stylistically diverse data collections [46,61], showed reduced performance, especially in prediction (Table 2). It seems that in such cases, custom-build CNN models may enhance validation accuracy, as demonstrated by Justen et al. [46], where identification accuracy increased from 94% (ResNet50) to 99.10% (custom CNN model).

The confusion matrix (Figure 11) shows that most classification errors occurred between larvae and nymphs, which share some visual similarities. Nevertheless, all images in our dataset included a visible size scale, and significant morphological differences-body size and the number of leg pairs (three in larvae versus four in nymphs)—were clearly distinguishable. These misclassifications may likely originate from suboptimal tick positioning, partial occlusion of key structures, visual similarity between classes, or image artifacts such as shadows and reflections—particularly visible in high-magnification images of larvae. Adult stages were classified with higher reliability, with only 2% of misclassifications occurring between males and females. These findings suggest that even in high-quality, standardized images, certain developmental stages remain more challenging to distinguish and may benefit from future inclusion of morphometric parameters.

In practical terms, the created model can serve as an auxiliary tool in healthcare service, potentially reducing the time spent on insect identification and eliminating the need for qualified entomological knowledge to visually assess the developmental stage of the tested individuals. The presented solution can potentially be implemented as a mobile application. An American smartphone tool, the “TickPhone App” [68], was developed and intended for use by untrained healthcare individuals, as Butler et al. pointed out a real issue with tick identification among primary care providers [69]. The application utilizes a neural network trained to recognize the three most common tick species in North America, which limits its effectiveness in Europe due to different tick fauna [1].

This work represents one stage in the analysis of the tick fauna of Zabrze, south Poland. In the next step, the collected specimens will be assessed for the prevalence of *Borrelia burgdorferi* spirochetes. Combining laboratory results with computer vision methods may help determine whether a neural network can detect significant morphological differences between individuals infected with *Borellia* spirochetes and those that are not.

## 6. Conclusions

The identification of tick species and their developmental stages is important in the context of managing tick-borne diseases. This study presents an innovative solution utilizing Convolutional Neural Networks (CNNs) to identify the developmental stages of *Ixodes ricinus*, one of the most common tick species in Europe. This is the first application of CNN models to European tick fauna, whereas previous research has focused on species from North America. The CNN model developed for classifying the developmental stages of *Ixodes ricinus* achieved 95% accuracy after 30 epochs of training on RGB images, which increased to 98% when using higher-resolution images (512 × 512 pixels). The Grad-CAM visualizations identified the dorsal shield and specific body regions as key features for classification, and the model maintained high accuracy even on grayscale images. Problematic for the model are misclassifications between similar categories (e.g., ‘larva’ vs. ‘nymph’) and lower effectiveness on images with complex backgrounds or distant ticks. Overall, the model demonstrated high stability and potential for improvement with extended training data, including a large number of photos of engorged ticks, which exhibit significant morphological changes.

## Figures and Tables

**Table 1:** Datasets Summary—Databases.

ID	Dataset Size	Image Characterization	Image Res.	Tick Species
1	12,000	microscopic, white background	3648 × 2736	*Amblyomma americanum*, *Dermacentor variabilis*, *Ixodes scapularis*
2	12,777	random internet-user captured	224 × 224	*Amblyomma americanum*, *Dermacentor variabilis*, *Ixodes scapularis*
3	2130	microscopic, white background	224 × 224	*Ixodes scapularis*, *Dermacentor variabilis*, *Amblyomma—americanum Haemaphysalis* sp.
4	1258	microscopic, white background	300 × 300	*Ixodes scapularis* vs. other species
5	1323	microscopic, white background	320 × 320 or 512 × 512	*Ixodes ricinus*

**Table 2:** Datasets Summary—Model Efficiency.

ID	Network Architecture	Validation Accuracy	Prediction Accuracy	Reference
1	VGG	99.37%	—	[59]
	ResNet50	99.42%	—	
	InceptionV3	99.50%	—	
	DenseNet121	99.20%	—	
	MobileNet121	98.73%	—	
2	InceptionV3	92.00%	87.80%	[46]
3	ResNet50	94.00%	75.00%	[60]
	Custom model	99.10%	80.00%	
4	Inception-ResNet	92.04%	—	[61]
	Custom model	91.68%	—	
5	Xception	95.00%	—	this work
	Xception (High Resolution Images)	98.00%	—	

## Data Availability

The original contributions presented in this study are included in the article. Further inquiries can be directed to the corresponding author.
